# Supplementary material for: Development of a Transcriptional Factor PuuR-Based Putrescine-Specific Biosensor in Corynebacterium glutamicum
Source: Bioengineering (Basel). 2023 Jan 24;10(2):157. doi: 10.3390/bioengineering10020157 (PMC9951944; doi:10.3390/bioengineering10020157)
Supplement: Supplementary file 1 [file bioengineering-10-00157-s001.zip › bioengineering-2121843-supplementary.pdf]

bioengineering

Development of a transcriptional factor PuuR-based putrescine-specific biosensor in *Corynebacterium glutamicum*

Nannan Zhao [1,2,3], Jian Wang [3], Aiqing Jia [3], Ying Lin [1,2] and Suiping Zheng [1,2,*]

1 Guangdong Key Laboratory of Fermentation and Enzyme Engineering, School of Biology and Biological Engineering, South China University of Technology, Guangzhou, 510006, China
2 Guangdong research center of Industrial Enzyme and Green Manufacturing Technology, School of Biology and Biological Engineering, South China University of Technology, Guangzhou, 510006, China
3 Animal Husbandry and Fisheries Research Center of Guangdong Haid Group Co.,Ltd, Guangzhou, 511400, China
* Correspondence: spzheng@scut.edu.cn; Tel: +86 13822153344

1. Supplementary tables

Table S1. Bacterial strains used in this study and their characteristics.

Strains and plasmids	Description	Source
**Strains**		
E. coli TOP 10	F⁻*mcr*A Δ(*mrr-hsd*RMS-*mcr*BC) φ80 *lac*ZΔ*M15* Δ*lac*X74 *rec*A1 *ara*Δ139 Δ(*ara-leu*)7697 *gal*U *gal*K *rps*L (Str^R) *end*A1 *nup*G	Invitrogen
Cg14067	*Corynebacterium glutamicum* ATCC 14067	ATCC
PUTP	The promoter of *argCJBD* in the Cg14067 genome was replaced with a strong P*eftu* promoter. Then, *E. coli*-derived SpeC was overexpressed.	This study
PUTP△	The *argF* gene was knocked out from the PUTP genome of the strain	This study
PUTP△D	Based on the recombinant strain PUTP△, the 7th codon of *snaA* on the genome was change to TAA	This study
**Plasmids**		
pEC-XK99E	Km^r, shuttle expression vector of *E. coli*/*C. glutamicum*	(Kirchner and Tauch 2003)
pEC-T18-mob2	Tet^r, shuttle expression vector of *E. coli*/*C. glutamicum*	(Kirchner and Tauch 2003)
pEC-XC99E-recET	Cm^r, pEC-XC99E derivative, expressing the recE and recT genes	(Huang et al. 2017)
T18-PpuuAF-mCherry	Tet^r, pEC-T18-mob2 derivative, expressing the *mCherry* gene under control of the P*puuA*F promoter of *E. coli*	This study
T18-PpuuAS-mCherry	Tet^r, pEC-T18-mob2 derivative, expressing the *mCherry* gene under control of the P*puuA*S promoter of *E. coli*	This study
T18-PpuuDF-mCherry	Tet^r, pEC-T18-mob2 derivative, expressing the *mCherry* gene under control of the P*puuD*F promoter of *E. coli*	This study
T18-PpuuDS-mCherry	Tet^r, pEC-T18-mob2 derivative, expressing the *mCherry* gene under control of the P*puuD*S promoter of *E. coli*	This study
pEC-XK99E-PuuR	Km^r, pEC-XK99E derivative, expressing the PuuR of *E. coli*	This study
pSenPuuREGFP	Tet^r, pEC-T18-mob2 derivative, encodes PuuR of *E. coli*	This study

	under the control of promoter P*trc*, and its response promoter of P*puuD*F with a transcriptional fusion to *egfp*	
pSenPuuRsfGFP	Tet[r], pEC-T18-mob2 derivative, encodes PuuR of *E. coli* under the control of promoter P*trc*, and its response promoter of P*puuD*F with a transcriptional fusion to *sfgfp*	This study
pSenPuuRsfGFP-Psod	Tet[r], pEC-T18-mob2 derivative, encodes PuuR of *E. coli* under the control of promoter P*sod*, and its response promoter of P*puuD*F with a transcriptional fusion to *sfgfp*	This study
pSenPuuRsfGFP-PcspB	Tet[r], pEC-T18-mob2 derivative, encodes PuuR of *E. coli* under the control of promoter P*cspB*, and its response promoter of P*puuD*F with a transcriptional fusion to *sfgfp*	This study
pSenPuuRsfGFP-Pddh	Tet[r], pEC-T18-mob2 derivative, encodes PuuR of *E. coli* under the control of promoter P*ddh*, and its response promoter of P*puuD*F with a transcriptional fusion to *sfgfp*	This study
pSenPuuRsfGFP-PaspB	Tet[r], pEC-T18-mob2 derivative, encodes PuuR of *E. coli* under the control of promoter P*aspB*, and its response promoter of P*puuD*F with a transcriptional fusion to *sfgfp*	This study
pEC-XK99E-SpeC	Km[r], pEC-XK99E derivative, encodes ornithine decarboxylase SpeC	This study

Table S2. Primers used in this study and their sequences.

**Primers**	**DNA sequences (5′-3′)**
99E-S	GGCTGTTTTGGCGGATGAGAGA
99E-A	CATGGTCTGTTTCCTGTGTGAAA
puuR-99E-S	CACAGGAAACAGACCATGATGAGTGATGAGGGACTGGCGC
puuR-99E-A	TCATCCGCCAAAACAGCCTTAAAACGTGGTGGGCGTATGG
XK99E-JD-A	TTGCTTCGCAACGTTCAAATCCGC
PEC-XK-99E-JD-S	TTGACAGCTTATCATCGACTGCAC
T18-A	ATTGCGTTGCGCTCACTGCCCGCT
T1T2-S	CCCATGCGAGAGTAGGGAACTGCC
mCherry-S	ATGGTTTCCAAGGGCGAGGAGGACA
mCherry-T1T2-A	TTCCCTACTCTCGCATGGGTTACTTGTAGAGTTCGTCCATGCC
PpuuAS-mCherry-A	CCTCGCCCTTGGAAACCATGATTCTTCGCCTTTGGTTTGTT
PpuuAS-T18-S	GCAGTGAGCGCAACGCAATGTTTTCATTTTTGCAAACTCAAT
PpuuAF-T18-S	GCAGTGAGCGCAACGCAATCCGGATTGTTCATTATATTTTCCA
PpuuDF-T18-S	GCAGTGAGCGCAACGCAATGATTCTTCGCCTTTGGTTTGTTTT
PpuuDF-mCherry-A	CCTCGCCCTTGGAAACCATACGACACCGATAACCGGATTGTTC
PpuuDS-T18-S	GCAGTGAGCGCAACGCAATTCTGTATGCAAACTAAATGTTTGT
PpuuDS-mCherry-A	CCTCGCCCTTGGAAACCATGCTCAATCTCACAAAGTGGACTA
T18-JD-S	TTTGAGTGAGCTGATACCGCTCGC

T18-JD-A	TTTGATGCCTGGCAGTTTATGGC
mCherry-JD-A	ATGATTGCCATGTTGTCCTCCT
PuuR-T2-RP4-S	CACCAAGGAAAGTCTACAAAAAGGCCATCCGTCAGGATGGC
PuuR-trc-T18-A	ATTTTGCCAAAGGGTTCGTTGACAATTAATCATCCGGCTCG
T18-trc-S	CGAACCCTTTGGCAAAATCCTG
T18-RP4-A	TGTAGACTTTCCTTGGTGTATCC
T1T2-EGFP-S	AAGTAACCCATGCGAGAGTAGGGAACTGCC
PpuuD-A	ACGACACCGATAACCGGATTGTTC
EGFP-PpuuD-full-A	AATCCGGTTATCGGTGTCGTATGAGCAAGGGCGAGGAGCTGT
EGFP-T1T2-A	TTCCCTACTCTCGCATGGGTTACTTGTACAGCTCGTCCATGC
T1T1-sfGFP-S2	AATAACCCATGCGAGAGTAGGGAACTGC
sfGFP-PpuuD-S	ATCCGGTTATCGGTGTCGTATGAGCAAAGGAGAAGAACTTTTC
sfGFP-T1T2-A	TTCCCTACTCTCGCATGGGTTATTTGTAGAGCTCATCCATGC
EGFP-JD-A	ATGTGGTCGGGGTAGCGGCTGAA
sfGFP-JD-A	TAGTGCGTTCCTGTACATAACCT
PT18-JD-A	TCGGTGCGGGCCTCTTCGCTATTA
PUUR-JD-S	AGCTTCATTGACACACCCTGA
ORI-JD-A	GCGGTGGTTTTTTTGTTTGCA
puuR-A	ATGAGTGATGAGGGACTGGCG
T18-trc-S	CGAACCCTTTGGCAAAATCCTG
sod-puuR-S	CGCCAGTCCCTCATCACTCATGGGTAAAAAATCCTTTCGTAG
sod-t18-A	GATTTTGCCAAAGGGTTCGTAGCTGCCAATTATTCCGGGC
PcspB-puur-S	CCAGTCCCTCATCACTCATAGAGGCGAAGGCTCCTTGAATA
PcspB-T18-A	GATTTTGCCAAAGGGTTCGAATTCCTGTGAATTAGCTGAT
ddh-puur-S	GCCAGTCCCTCATCACTCATGTTCTTGTAATCCTCCAAAATTG
ddh-t18-A	GATTTTGCCAAAGGGTTCGGGGTGTTTTCATCCAAACCCAA
aspB-puur-S	CGCCAGTCCCTCATCACTCATAACTGCGTACCTCCGCATGT
aspB-t18-A	GATTTTGCCAAAGGGTTCGAGCTAGAGTTATGCGAAGGATC
argC-U-S	CCTCGCGCAGTTTTGTTGGGTAAT
argC-U-loxp71-A	GCAGTATAACTTCGTATAATGTATGCTATACGAACGGTAC-GGGTTAGACATGCAAAAACCC
C-K-loxp71	TACCGTTCGTATAGCATACATTATACGAAGTTATACTG-CATAATTCGTGTCGCTCA
C-K-loxp66	TACCGTTCGTATAATGTATGCTATACGAAGTTA-TATGGGTTAAAAAGGATCGATCC
Peftu-loxp66-S	CCCATATAACTTCGTATAGCATACATTATACGAACGGTAGGG-TAGCTGGTAGTTTGAAAATC
Peftu-PargC-D-A	GATTGCAACCTTGATTGTCATTGTATGTCCTCCTGGACTTCGTG
PargC-D-S	ATGACAATCAAGGTTGCAATCGC

PargC-D-A	GTGCACCTTCTGGAAGAACATGC
PargC-JD-S	TTCCGCAGTTGAAGAGCCAGCAGT
PargC-JD-A	GGAATGTGTGGCATCAATTCACCG
argF-U-S	GGTGAAACGGGCGTTGTTCCAG
argF-U-loxp71	TGCAGTATAACTTCGTATAATGTATGCTATACGAACGGTAC-CGCGTCGAAGGAGAAGCGAGTA
argF-D-loxp66	ACCCATATAACTTCGTATAGCATACATTATACGAACGGTACCGA-TACCTGGGTATCCATGGG
argF-D-A	AAGCCTTGAACTAGGGGCGCTT
△argF-JD-S	GCTATTGCCGAGACAATCGCATA
△argF-JD-A	TCTGAGCGTGGAGGCGGTTTTCT
PJYS2-snaA-S	CTACTGTTGTAGATCCTGCTACACAAGCTGACTTCCCTATTTAAA-TAAAACGAAAGGCT
PJYS2-snaA-A	CGTTTTATTTAAATAGGGAAGTCAGCTTGTGTAGCAG-GATCTACAACAGTAGAAATTCG
PJYS2-snaA-crR-JD-S	AGGGAAGTCAGCTTGTGTAGCAGG
pJYS2-JD-A	ATCCTGGATGCCCGTTCTCAAT
pJYS1-JD-A	TTCGCGCGCGAATTGCAAGCTGAT
pJYS1-JD-S	GTAAGCATGATCTCAATGGTTCG
O-snaA	TCGACGATCTTAGGGAAGTCAGCTTGTGTAGCTTACAAAAC-GGTGGGACTCATGGATAC
DsnaA-JD-S	TTGATGCTTCTACCTAGCTCAAG
DsnaA-JD-A	AGTCCCAAAATTGAGACCGTCCA

Table S3. Percent identity matrix of candidate protein sequences.

ODCs	Protein No.	Percent identity					
ODC1	WP_006819050.1	100.00	66.29	68.63	84.70	80.25	88.04
ODC2	WP_152654430.1	66.29	100	62.91	66.29	66.43	65.87
ODC3	WP_064601238.1	68.63	62.91	100	68.63	68.07	68.78
ODC4	WP_013097558.1	84.70	66.29	68.63	100	81.78	84.84
ODC5	WP_016535388.1	80.25	66.43	68.07	81.78	100	78.72
ODC6	EAP4732819.1	88.04	65.87	68.78	84.84	78.72	100

2. Supplementary figures

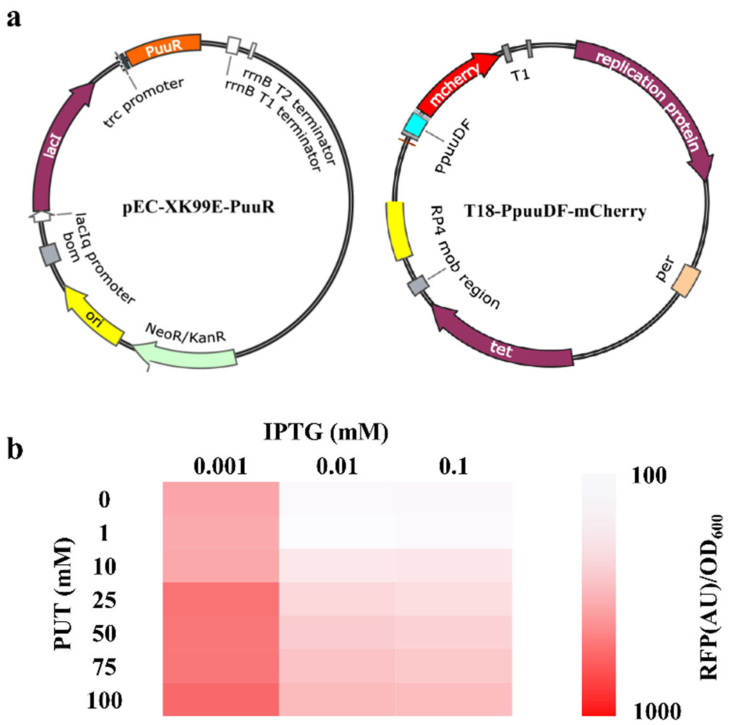

Figure S1. Testing of the two-plasmid biosensor system. (a) Diagram of the two-plasmid biosensor system used for detecting PuuR putrescine sensing; (b) Checkerboard assay of the two-plasmid PuuR biosensor system. The Y-axis is putrescine concentration (mM), and the X-axis is IPTG concentration (mM). The color bar on the right shows the fluorescence ratio. IPTG: isopropyl-β-D-thio-galactoside

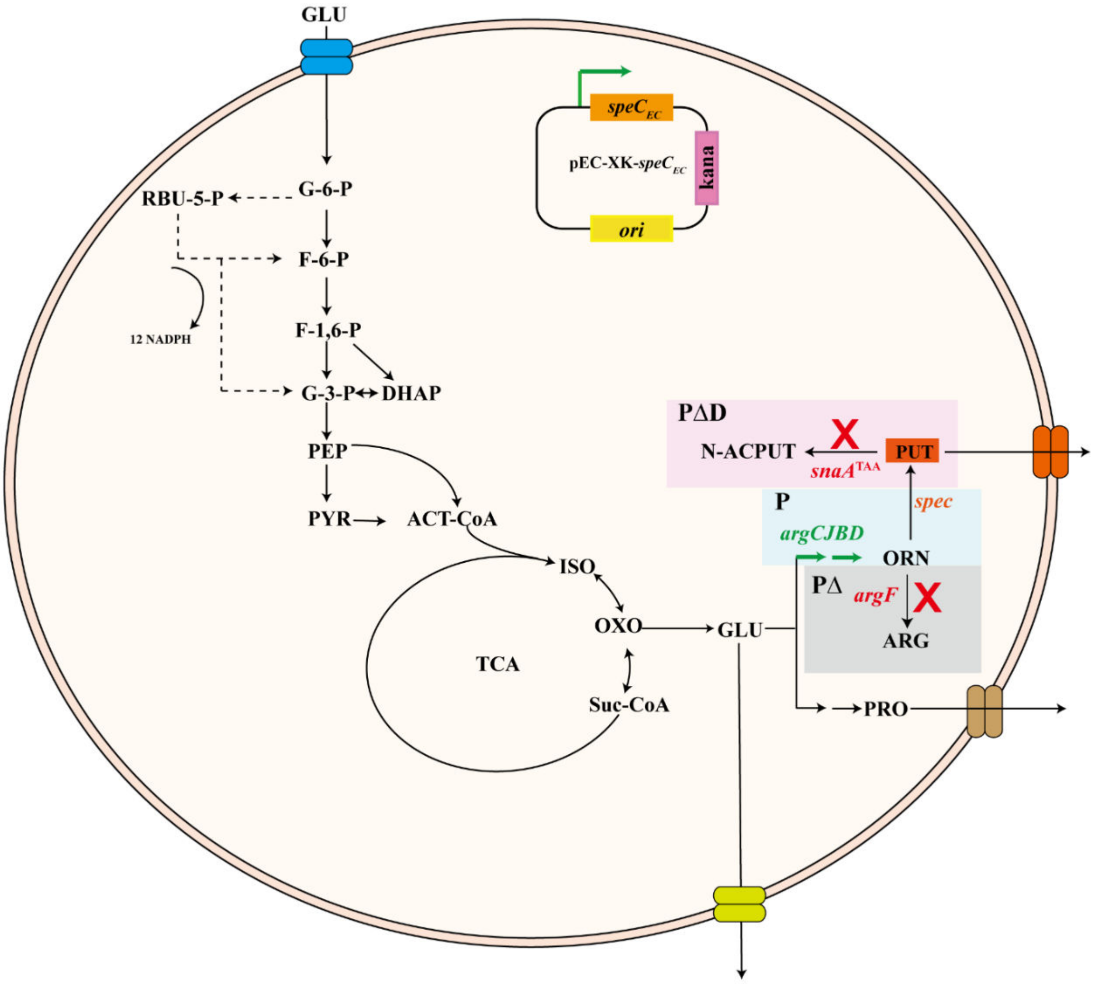

Figure S2. Schematic diagram of the metabolic engineering strategy for putrescine biosynthesis.

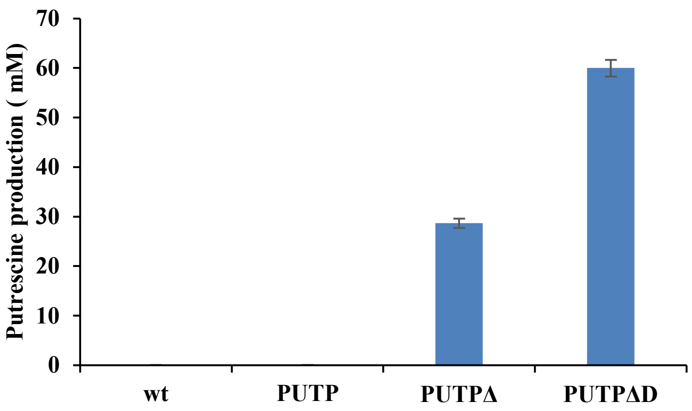

Figure S3. Putrescine production by recombinant strains. Values are presented as the mean ± standard deviation of three independent experiments.

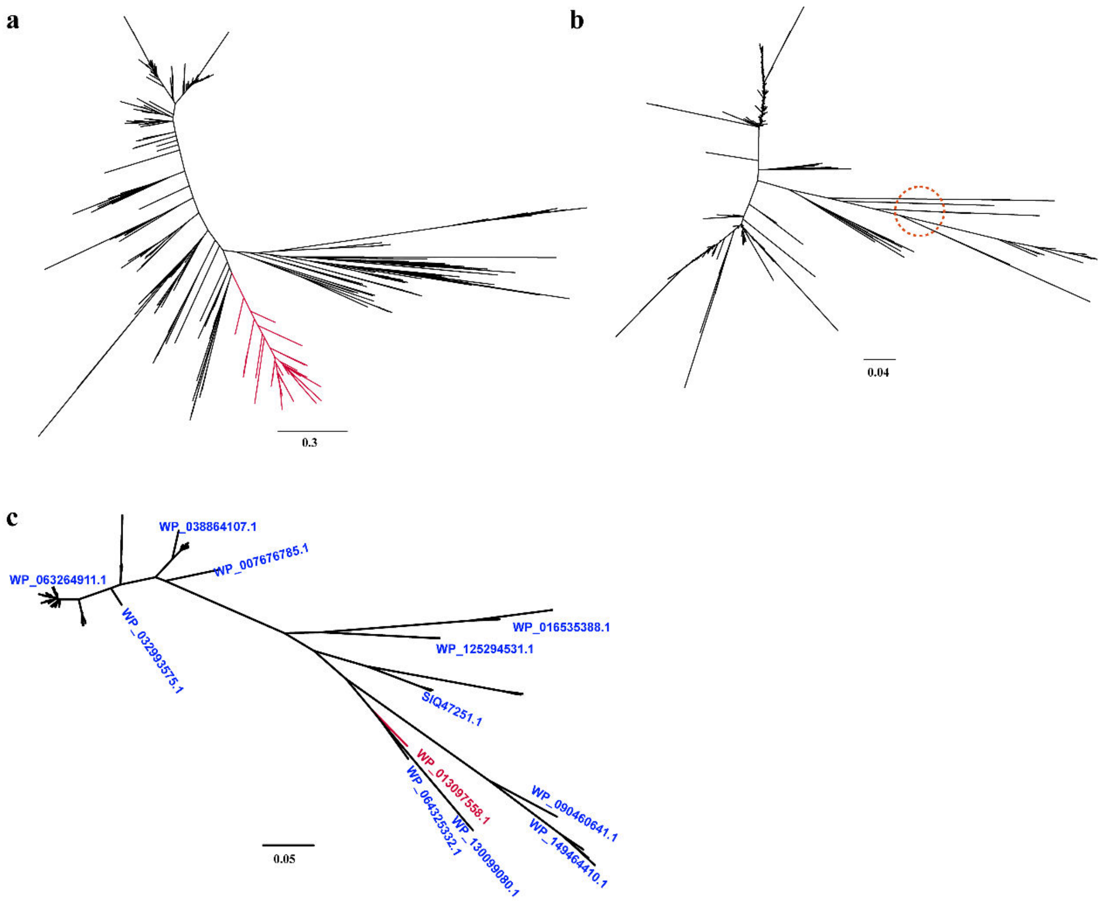

Figure S4. Phylogenetic analysis of ODCs with SpeF$_{\mathrm{ECL}}$ as a reference. (a) Phylogenetic tree constructed from representatives of 8245 ornithine decarboxylase sequences with approximately 50%–90% similarity with SpeF$_{\mathrm{ECL}}$; (b) Phylogenetic analysis of 1805 available members in the SpeF$_{\mathrm{ECL}}$ subbranch; (c) Phylogenetic tree analysis of ODC sequence of the SpeF$_{\mathrm{ECL}}$ subbranch. SpeF$_{\mathrm{ECL}}$ (red circles) is in the subbranch containing 124 ODC sequences. ODC: ornithine decarboxylase.

References

1. Huang Y, Li L, Xie S, Zhao N, Han S, Lin Y, Zheng S (2017) Recombineering using RecET in *Corynebacterium glutamicum* ATCC14067 via a self-excisable cassette. Scientific Reports 7(1):7916. https://doi.org/10.1038/s41598-017-08352-9

2. Kirchner O, Tauch A (2003) Tools for genetic engineering in the amino acid-producing bacterium *Corynebacterium glutamicum*. Journal of biotechnology 104(1-3):287-299. https://doi.org/10.1016/S0168-1656(03)00148-2
